# Cell Polarity, Epithelial-Mesenchymal Transition, and Cell-Fate Decision Gene Expression in Ductal Carcinoma In Situ

**DOI:** 10.1155/2012/984346

**Published:** 2012-04-02

**Authors:** Danila Coradini, Patrizia Boracchi, Federico Ambrogi, Elia Biganzoli, Saro Oriana

**Affiliations:** ^1^Department of Work Medicine “Clinica del Lavoro L. Devoto”, Section of Medical Statistics and Biometry “G.A. Maccacaro”, University of Milano, 20133 Milan, Italy; ^2^Senology Center, Casa di Cura Ambrosiana, Istituto Sacra Famiglia, Cesano Boscone, 20090 Milano, Italy

## Abstract

Loss of epithelial cell identity and acquisition of mesenchymal features are early events in the neoplastic transformation of mammary cells. We investigated the pattern of expression of a selected panel of genes associated with cell polarity and apical junction complex or involved in TGF-*β*-mediated epithelial-mesenchymal transition and cell-fate decision in a series of DCIS and corresponding patient-matched normal tissue. Additionally, we compared DCIS gene profile with that of atypical ductal hyperplasia (ADH) from the same patient. Statistical analysis identified a “core” of genes differentially expressed in both precursors with respect to the corresponding normal tissue mainly associated with a terminally differentiated luminal estrogen-dependent phenotype, in agreement with the model according to which ER-positive invasive breast cancer derives from ER-positive progenitor cells, and with an autocrine production of estrogens through androgens conversion. Although preliminary, present findings provide transcriptomic confirmation that, at least for the panel of genes considered in present study, ADH and DCIS are part of a tumorigenic multistep process and strongly arise the necessity for the regulation, maybe using aromatase inhibitors, of the intratumoral and/or circulating concentration of biologically active androgens in DCIS patients to timely hamper abnormal estrogens production and block estrogen-induced cell proliferation.

## 1. Introduction

Ductal carcinoma in situ (DCIS), also known as intraductal carcinoma, is the most common type of noninvasive breast cancer in women [1]. From the mid-1970s, the incidence of DCIS has sharply increased, primarily because of the adoption of radiographic screening for invasive carcinoma. Currently, it accounts for approximately 25% of newly diagnosed breast cancer cases [1].

DCIS is a nonobligate precursor to invasive breast cancer, and for this reason some members of the 2009 US National Institutes of Health DCIS consensus conference proposed to remove the word “carcinoma” from the term DCIS [2]. Nevertheless, experimental studies have shown the presence of carcinoma precursor cells in DCIS lesions [3–5], and clinical evidence indicates that approximately 50% of cases will progress to invasive breast cancer if untreated [6, 7]. As a result, the malignant nature of DCIS remains debated, primarily because of the limited knowledge of DCIS arising and development. In fact, while several genome-wide studies have compared the gene profiles of DCIS and invasive breast cancer, very few studies have investigated and recognized the molecular alteration that characterize DCIS with respect to normal tissue [8–11].

DCIS is defined as an abnormal proliferation of transformed mammary epithelial cells within the closed environment of a duct, likely in response to microenvironment alterations including hypoxia and nutrient deprivation [4, 12]. Among the processes early affected during mammary cells transformation, those involved in the establishment and maintenance of epithelial cell identity and tissue specificity are of particular relevance. In fact, epithelial mammary cells are characterized by an asymmetric distribution of cytoplasmic and membrane proteins, termed apicobasolateral cell polarity, essential for a correct cell-cell adhesion and the formation of an epithelial sheet. As a result of an epithelial to mesenchymal transition (EMT), during neoplastic transformation, cell polarity and epithelial morphology are early lost polarized and immotile epithelial cells acquire a fibroblast-like morphology and increased cell motility [13].

EMT process can be induced by a variety of signaling pathways among which the main and best characterized is that involving transforming-growth factor-*β* (TGF-*β*). TGF-*β* is a multifunctional cytokine and a powerful tumor suppressor that governs many aspects of mammary epithelial cells physiology and homeostasis [14]. Under abnormal microenvironment conditions; however, some mammary epithelial cells may acquire resistance to TGF-*β*, circumvent its cytostatic effect and tumor suppressive activity, and activate EMT [15, 16].

Recent studies have demonstrated that EMT may generate cells with stemness-like properties, especially in the transitioning mammary epithelial cell compartment [17, 18]. Therefore, a interrelationship among EMT (TGF-*β* mediated), disruption of the mechanisms deputed to cell polarity and adhesion control, and acquisition of stemness-like features can be assumed already in DCIS [19, 20].

Taking advantage from the only microarray dataset publicly accessible at the ArrayExpress web site, we investigated the pattern of expression of a selected panel of genes involved in TGF-*β*-mediated EMT or associated with epithelial cells identity (i.e., cell polarity and apical junction complex) and cell-fate decision in a series of DCIS and corresponding patient-matched histologically normal (HN) epithelium [11]. As the whole-gene expression profile of patient-matched atypical ductal hyperplasia (ADH) was also available, we further compared DCIS and ADH profiles to verify the hypothesis according to which breast cancer progression is a multistep process involving a continuum of changes from normal phenotype through hyperplastic lesions, carcinoma in situ, and invasive carcinoma [21].

## 2. Materials and Methods

### 2.1. Materials

As reported in the original paper [11], patient-matched samples (HN, ADH, and DCIS) were isolated via laser capture microdissection from surgical specimens of 12 preoperative untreated patients with an ER-positive (immunohistochemically evaluated) sporadic breast cancer. Gene expression was determined by using the Affymetrix Human Genome HG-U133A GeneChip; corresponding microarray dataset was publicly available at the ArrayExpress web site (http://www.ebi.ac.uk/arrayexpress/) with the Accession number E-GEOD-16873.

### 2.2. Gene Selection

To select the panel of genes specifically involved in the TGF-*β*-activated EMT, cell polarity and apical junction complex, and cell-fate decision, we combined Gene Ontology (http://www.geneontology.org) and PubMed (http://www.ncbi.nlm.nih.gov) information. In addition, since cancer cells often increase their autocrine production of TGF-*β* to activate angiogenesis in response to oxygen and nutrients deprivation [14], we also included some genes coding for angiogenesis-inducing factors. On the whole, a set of 199 genes evolutionarily conserved in *Homo sapiens* was established (see Supplementary Table  1 available on line at doi 10.1155/2012/984346). However, because 27 genes had no corresponding probe-sets on the HG-U133A GeneChip, the gene set was actually composed of 172 elements, 33 of which involved in EMT activation [13–15, 21–23], 75 involved in cell polarity and apical junction assembly [24–26], 36 involved in cell fate-decisions and in the maintenance of a self-renewal state in tumorigenic adult tissues [27–29], 28 involved in hormone steroid signaling [30–32], angiogenesis activation [33, 34] or used as luminal and basal markers [35–38]. These 172 genes corresponded to 339 Affymetrix probe-sets, as verified by GeneAnnot system v2.0 (http://bioinfo2.weizmann.ac.il/geneannot/), that additionally provided us information about the quality of each probe-set in terms of sensitivity and specificity score [39] (see Supplementary). 

### 2.3. Statistical Analysis

As some genes are recognized by more than a single probe set, each of which characterized by an individual specificity and sensitivity that differently contribute to gene expression value, a gene expression mean value was calculated after weighting each probe-set for its own sensitivity and specificity score. Specifically, each expression value (already log 2 transformed in the original dataset) was multiplied for the semi sum of sensitivity and specificity scores of the corresponding probe set. Given the patient-matched samples study design, all statistical analyses were performed considering a regression model for repeated measures with random effect, and the differential gene expression was evaluated by *t*-test on regression coefficients. To correct for multiple testing, the false discovery rate (FDR) was used [40].

To evidence latent variables accounting for genes correlations, a factor analysis was applied [41] in the following three comparisons: DCIS and paired ADH, DCIS and paired HN, and ADH and paired HN. The number of retained factors was selected according to the scree test [42]. To facilitate the interpretation of the factors, varimax rotation was applied. Loading values lower than 0.3 were not considered.

All analyses were performed using open source software R 2.11.1 packages HDMD (http://www.R-project.org).

## 3. Results and Discussion

Genes found differentially expressed (*P* < 0.05) between DCIS and NH or ADH and NH are reported in Table 1. Specifically, 47 of the 172 selected genes were found differentially expressed between DCIS and NH (11 with an estimated FDR < 0.01) and 28 were found differentially expressed between ADH and NH (only one with an estimated FDR < 0.01). Notably, 24 of the 28 genes found differentially expressed between ADH and NH were found differentially expressed (in a similar manner) also between DCIS and NH. The persistence of this “core” of genes, dysregulated in a similar manner in both invasive breast cancer precursors, seems to support the hypothesis of ADH as the direct precursor of DCIS. In fact, in agreement with the proposed multistep process, DCIS showed an increased number of genes differentially expressed with respect to ADH.

With respect to normal tissue, both DCIS and ADH showed the overexpression of *ESR1*, coding for the estrogen receptor; *CD24,* coding for a mucin-like cell-adhesion molecule positively associated with a terminally differentiated luminal phenotype [43, 44]; *GATA3,* coding for a transcription factor involved in mammary gland morphogenesis [35, 36]; *CLDN7*, *EPCAM,* and *TJP3*, coding for tight junction components; *CDC42* and *TIAM1*, coding for two small GTPase family members involved in cell polarity and apical junction complex formation. Concomitantly, both breast cancer precursors showed the underexpression of *EGFR* gene, in which expression is generally negatively associated with *ESR1 *expression, *KRT5*, *KRT14,* and *KRT17*, all coding for cytokeratins associated with a basal phenotype. On the whole, this pattern of expression clearly indicates that DCIS and ADH are both characterized by a terminally differentiated luminal phenotype. Since all specimens were derived from patients with an ER-positive ductal carcinoma, it is conceivable the hypothesis that the establishment of an estrogen-dependent phenotype, in response to estrogens present in the microenvironment, should be a very early event in the tumorigenic process. Such a finding is in agreement with the model for breast cancer development proposed by Dontu et al. [45], according to which ER-positive cancers should derive from transiently amplifying ERpositive progenitor cells. Escaped from proliferation control as a consequence of genetic and epigenetic alterations in genes involved in cell-fate decision, these ER-positive progenitor cells should generate cells constitutively expressing estrogen receptor. Once established in ADH, this terminally differentiated luminal phenotype seems to consolidate in DCIS as demonstrated by the presence, among the genes exclusively expressed in DCIS, of *KRT18, KRT19 *(coding for some luminal-associated cytokeratins) and *DLG1, DLG3, *and* MPP5 *(coding for some cell polarity complex components). 

With respect to both tumor precursors, histologically normal tissue expressed genes coding for some transcription factors involved in EMT (*SNAI2 *and* TGFBR3*) and cell-fate decision (*FOXC1 *and* JAG2), *and for stemness-associated features (*ABCG2*). This finding is more evident considering the 47 genes differentially expressed between DCIS and normal tissue among which we found some other genes involved in the TGF-*β*-mediated EMT (*AKT3, ID4 *and* TGFBR2*), and cell-fate decision and self-renewal (*NOTCH4 *and* PROM1*).

Such an apparently paradoxical finding, that is, the positive association between histologically normal tissues and EMT- and stemness-related genes (more likely expected in transformed tissues) is not surprising since we observed a similar behavior in an independent cases series composed of primary breast cancers and corresponding patient-matched normal tissue (submitted paper) and in normal pleura with respect to pleural mesothelioma [46]. In agreement with the physiological remodeling of the mammary gland, such a finding should support the persistence of resident stem/progenitor cells in normal tissue [47, 48].

**Figure 1:**
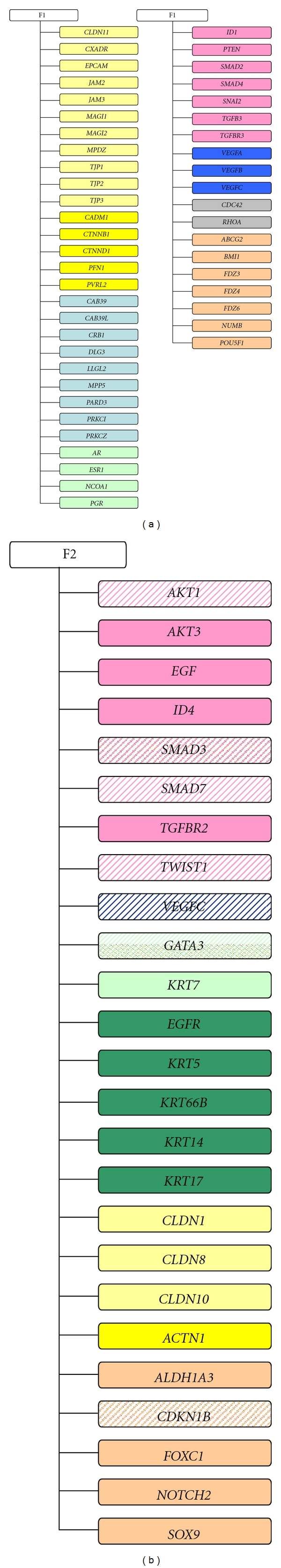
Factor analysis in DCIS subgroup. Schematic representation of genes with a loading value <|0.6| characterizing the first (F1) and the second factor (F2). Solid color indicates a positive loading value whereas dashed color indicates a negative loading value. Color correspondence: *light yellow*, tight junction components; *dark yellow*, adherens junction components; *light blue*, polarity complexes components; *dark blue*, angiogenesis; *orange*, cell-fate decision; *light green*, luminal markers and hormone steroid; *dark green*, basal markers; *pink*, epithelial-mesenchymal transition, *grey*, GTPase family members.

**Figure 2:**
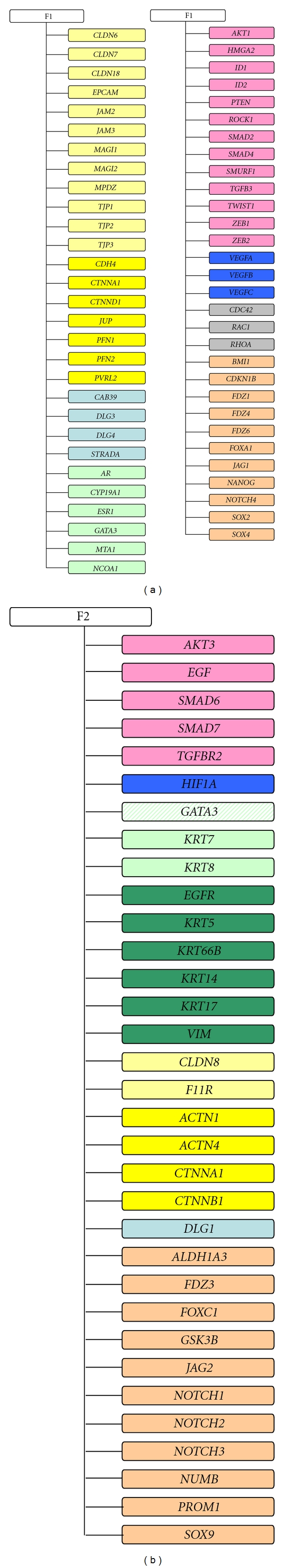
Factor analysis in ADH subgroup. Schematic representation of genes with a loading value <|0.6| characterizing the first (F1) and the second factor (F2). Solid color indicates a positive loading value whereas dashed color indicates a negative loading value. Color correspondence: *light yellow*, tight junction components; *dark yellow*, adherens junction components; *light blue*, polarity complexes components; *dark blue*, angiogenesis; *orange*, cell-fate decision; *light green*, luminal markers and hormone steroid; *dark green*, basal markers; *pink*, epithelial-mesenchymal transition, *grey*, GTPase family members.

Factor analysis, applied to investigate the latent variables intrinsically associated with the selected 172 genes, corroborated these findings and highlighted some other interesting interrelations. In agreement with the results provided by *t*-paired test, factor analysis indicated that in both precursors (Figure 1(a) for DCIS and Figure 2(a) for ADH), the first factor (F1) was principally characterized by genes associated with an estrogen-dependent epithelial phenotype. In fact, within the genes with a positive loading value on F1, we found those coding for hormone steroid receptors (*AR *and* ESR1*) and transcriptional coactivator for steroid and nuclear hormone receptors (*NCOA1*), for tight (*EPCAM, MAGI1, MAGI2, MPDZ, TJP1, TJP2 *and* TJP3*) and adherens (*CTNND1, PFN1 *and* PVRL2*) junction components, and for small GTPase family members involved in epithelial cell polarization processes (*CDC42 *and *RHOA*). In addition, we found some genes associated with cell-fate decision: *BMI-1,* coding for a member of Polycomb group required to maintain the transcriptionally repressive state of many genes throughout embryo development [49] and adult tissues differentiation including mammary gland [50]; *FDZ4* and *FDZ6*, two members of the frizzled gene family involved in *β*-catenin-signaling transduction and intercellular transmission of polarity information in differentiated tissues [51].

Furthermore, in agreement with the notion that ER*α*, and TGF-*β*-signaling pathways are major regulators during mammary gland development [52], we found genes coding for TGF-*β* receptor* (TGFBR3)* or for proteins involved in canonical (*ID1, SMAD2 *and* SMAD4*) and noncanonical (*PTEN*, *RHOA *and* ROCK1*) TGF-*β* pathways [15, 16].

Finally, probably associated with the adaptation of DCIS and ADH to hypoxic stress caused by the unbalance between cell proliferation and oxygen supply, we also found *JAM2, JAM3, VEGFA*, *VEGFB, *and* VEGFC, *all genes involved in endothelial cell proliferation.

The second factor (F2) identified by factor analysis (Figure 1(b) for DCIS and Figure 2(b) for ADH) was conversely characterized by the positive loading value of several genes coding for mesenchymal markers (*EGF, EGFR, KRT5, KRT6B, KRT14 *and* KRT17*), and the negative loading value of *GATA3,* the gene coding for the transcription factor driving the luminal morphogenesis of the mammary gland [35, 36]. In addition, F2 was characterized by the presence of some genes coding for proteins playing a critical role in cell-fate decision and cell-renewal (*ALDH1A3, FOXC1, NOTCH2, PROM1, *and* SOX9*) [53].

Notably, when applied to ADH subgroup, factor analysis identified *CYP19A1 *as included in the panel of genes characterizing the first factor. This gene encodes for cytochrome P450 (better known as aromatase), the enzyme that catalyzes the conversion from circulating and rostenedione to estrone or testosterone to estradiol, and its presence should support the hypothesis of a very early activation of an autocrine production of estrogen. Furthermore, the concomitant presence of *CYP19A1, AR*, *ESR1,* and *NCOA1 *should provide a transcriptomic confirmation for the clinical evidence that high androgens level may have detrimental effect on breast carcinogenesis and progression due to a persistent local estrogen production that incessantly stimulates epithelial cell proliferation [54–56].

When applied to DCIS subgroup, factor analysis seems to indicate a consolidation of such an estrogen dependence as suggested by the additional presence of *PGR*, the gene coding for progesterone receptor and which expression is under estrogenic control.

## 4. Conclusions

Elucidating the initial steps of breast tumorigenesis is of paramount importance to allow an even early diagnosis and consequently an adequate treatment strategy aimed to prevent the malignant transformation of preneoplastic alterations. That is of particular importance for DCIS because of its high incidence [1] and facility in progressing to invasive breast cancer if untreated [6, 7].

Experimental evidence till now accumulated has clearly indicated that at the basis of the neoplastic transformation of mammary epithelial cells there are loss of apicobasal epithelial cell identity and acquisition of a functional mesenchymal morphology. Therefore, we investigated the pattern of expression of a selected panel of genes associated with epithelial cells identity (i.e., cell polarity and apical junction complex) or involved in TGF-*β*-mediated EMT and cell-fate decision in a series of DCIS and corresponding patient-matched normal tissue. In addition, we compared DCIS profile with that of patient-matched ADH to investigate the hypothesis according to which breast cancer progression is a multistep process involving a continuum of changes from normal phenotype through hyperplastic lesions, carcinoma in situ, and invasive carcinoma [21].

Statistical analysis seems to support this hypothesis because it identified a “core” of genes, mainly associated with a terminally differentiated luminal phenotype, and differentially expressed in both precursors with respect to the corresponding normal tissue. Notably, these alterations in gene expression did not result in a progressive mesenchymal transition but rather in a terminally differentiated luminal phenotype, in agreement with the model according to which ER-positive invasive breast cancer derives from ER-positive progenitor cells [45]. The constitutive expression of ER should make ADH and DCIS forming cells able to exploit the proliferative stimulus induced by estrogens whereas the establishment of an autocrine production of estrogens, through androgens conversion, should provide an additional selective advantage. The detrimental effect of such continuous estrogen stimulation should be corroborated by the observation that all patients included in the present study developed an invasive ER-positive ductal carcinoma.

Experimental evidence supporting the hypothesis that androgens conversion may be involved in DCIS development, and progression has been provided by a recent study in which the intratumoral concentration of estradiol and 5*α*-dihydrotestosterone (DHT), and the expression of some sex steroid-producing enzymes, including aromatase, has been evaluated in DCIS specimens [57]. The study clearly demonstrated that aromatase expression level was significantly higher in DCIS with respect to nonneoplastic tissue suggesting the self-sustaining process adopted by DCIS.

Taken together all these findings provide transcriptomic confirmation that, at least for the panel of genes considered in present study, ADH and DCIS are part of a tumorigenic multistep process and strongly arise the necessity for the regulation, maybe using aromatase inhibitors, of the intratumoral and/or circulating concentration of biologically active androgens in DCIS patients to timely hamper abnormal estrogens production and block estrogen-induced cell proliferation [58].

There is no doubt that present *in silico* study suffers for the limitation common to the majority of studies involving gene expression profile, that is, the lack of validation, at protein level, of the modulations observed at mRNA level. In fact, it is well known that mRNA transcript levels do not always reflect protein expression. However, the immunohistochemical data provided in The Human Protein Atlas largely confirms the differential gene expression that we observed between histologically normal and cancerous tissue. For instance, when we considered the 11 genes with an estimated FDR < 0.01 (Table 1, in bold), we found that protein expression of normal breast (glandular cell) and breast cancer tissue mainly paralleled the mRNA differential expression we observed in our dataset, making our preliminary findings more reliable and worthy of further investigation.

## Supplementary Material

Table 1: lists the 199 genes selected for the study.
Table 2: lists the 339 Affymetrix probe-sets corresponding to the 172 genes entered the study.

## Figures and Tables

**Table 1:** Differentially expressed genes between ADH or DCIS and histologically normal (HN) tissue (ordered according to *P* value).

ADH versus HN			DCIS versus HN		
Gene symbol	*P* value	Variation	Gene symbol	*P* value	Variation
**JAM3**	0.000045	↓	**JAM3**	0.000016	↓
JAG2	0.000337	↓	**EGFR**	0.000103	↓
CD24	0.000364	↑	**SNAI2**	0.000127	↓
SNAI2	0.000594	↓	**CLDN5**	0.000136	↓
EGFR	0.001897	↓	**JAM2**	0.000150	↓
FOXC1	0.002222	↓	**FOXC1**	0.000167	↓
EGF	0.003163	↓	**CD24**	0.000277	↑
ID2	0.003547	↑	**JAG2**	0.000297	↓
JAM2	0.005052	↓	**KRT5**	0.000420	↓
TJP3	0.005070	↑	**KRT14**	0.000425	↓
TGFBR3	0.005537	↓	**GATA3**	0.000490	↑
KRT17	0.005624	↓	TGFBR3	0.000710	↓
GATA3	0.005795	↑	TJP3	0.001046	↑
TP53	0.006737	↑	KRT17	0.001402	↓
CDH4	0.006916	↓	SOX4	0.001942	↑
AKT1	0.009044	↑	AKT1	0.002867	↑
CLDN7	0.012026	↑	CLDN8	0.003090	↓
EPCAM	0.015207	↑	CDC42	0.003643	↑
ABCG2	0.019210	↓	EGF	0.003752	↓
KRT5	0.019854	↓	EPCAM	0.004605	↑
KRT14	0.020117	↓	CLDN7	0.004749	↑
CLDN11	0.023005	↓	ESR1	0.007188	↑
PARD3	0.030202	↓	DLG1	0.007249	↑
SOX4	0.036127	↑	KRT19	0.007637	↑
CDC42	0.036404	↑	FOXA1	0.007844	↑
TIAM1	0.038034	↑	KRT18	0.010091	↑
ESR1	0.038527	↑	CDH3	0.010638	↓
PVR	0.039104	↓	TIAM1	0.010722	↑
			TGFBR2	0.013243	↓
			RHOA	0.013540	↑
			BRCA1	0.014098	↑
			ID4	0.017030	↓
			ID2	0.018115	↑
			NOTCH4	0.018550	↓
			PVRL2	0.019914	↑
			AKT3	0.020275	↓
			ACTN1	0.020619	↓
			PROM1	0.022238	↓
			CDH4	0.022933	↓
			DLG3	0.023798	↑
			F11R	0.031657	↑
			CTNNA1	0.033234	↑
			MTA2	0.033999	↓
			ABCG2	0.037966	↓
			MPP5	0.039023	↑
			HIF1A	0.039489	↑
			CDKN1A	0.047769	↑

In bold, genes with an estimated FDR < 0.01.
