# Supplementary material for: Cell Polarity, Epithelial-Mesenchymal Transition, and Cell-Fate Decision Gene Expression in Ductal Carcinoma In Situ
Source: Int J Surg Oncol. 2012 Apr 2;2012:984346. doi: 10.1155/2012/984346 (PMC3335180; doi:10.1155/2012/984346)
Supplement: Supplementary file 1 — Table 1: lists the 199 genes selected for the study. Table 2: lists the 339 Affymetrix probe-sets corresponding to the 172 genes entered the study. [file 984346.f1.doc]

Supplementary Table 1: Overall genes selected for the study

|  | *Official Gene Symbol* | *Gene Name* | *Entrez Gene ID* | *Entrez Gene Location* |
| --- | --- | --- | --- | --- |
| EMT activation | AKT1 | v-akt murine thymoma viral oncogene homolog 1 | 207 | 14q32.32 |
|  | AKT2 | v-akt murine thymoma viral oncogene homolog 2 | 208 | 19q13.1-q13.2 |
|  | AKT3 | v-akt murine thymoma viral oncogene homolog 3 | 10000 | 1q44 |
|  | EGF | Epidermal growth factor | 1950 | 4q25 |
|  | ERBB2 | V-erb-b2 erythroblastic leukemia viral oncogene homolog 2 (avian) | 2064 | 17q11.2-q12 |
|  | HMGA2 | High mobility group AT-hook 2 | 8091 | 12q15 |
|  | ID1 | Inhibitor of DNA binding 1, dominant negative helix-loop-helix protein | 3397 | 20q11 |
|  | ID2 | Inhibitor of DNA binding 2, dominant negative helix-loop-helix protein | 3398 | 2p25 |
|  | ID3 | Inhibitor of DNA binding 3, dominant negative helix-loop-helix protein | 3399 | 1p36.13-p36.12 |
|  | ID4 | Inhibitor of DNA binding 4, dominant negative helix-loop-helix protein | 3400 | 6p22.3 |
|  | PI3KCA | Phosphoinositide-3-kinase, catalytic, alpha polypeptide | 5290 | 3q26.3 |
|  | PTEN | Phosphatase and tensin homolog | 5728 | 10q23.3 |
|  | ROCK1 | Rho-associated, coiled-coil containing protein kinase 1 | 6093 | 18q11.1 |
|  | ROCK2 | Rho-associated, coiled-coil containing protein kinase 2 | 9475 | 2p24 |
|  | SIX1 | SIX homeobox 1 | 6495 | 14q23.1 |
|  | SMAD2 | SMAD family member 2 | 4087 | 18q21.1 |
|  | SMAD3 | SMAD family member 3 | 4088 | 15q22.33 |
|  | SMAD4 | SMAD family member 4 | 4089 | 18q21.1 |
|  | SMAD6 | SMAD family member 6 | 4091 | 15q21-q22 |
|  | SMAD7 | SMAD family member 7 | 4092 | 18q21.1 |
|  | SMURF1 | SMAD specific E3 ubiquitin protein ligase 1 | 57154 | 7q22.1 |
|  | SNAI1 | Snail homolog 1 (Drosophila) | 6615 | 20q13.1-q13.2 |
|  | SNAI2 | Snail homolog 2 (Drosophila) | 6591 | 8q11 |
|  | TCF3 | Transcription factor 3 (E2A immunoglobulin enhancer binding factors E12/E47) | 6929 | 19p13.3 |
|  | TGFB1 | Transforming growth factor, beta 1 | 7040 | 19q13.2 |
|  | TGFB2 | Transforming growth factor, beta 2 | 7042 | 1q41 |
|  | TGFB3 | Transforming growth factor, beta 3 | 7043 | 14q24 |
|  | TGFBR1 | Transforming growth factor, beta receptor I | 7046 | 9q22 |
|  | TGFBR2 | Transforming growth factor, beta receptor II | 7048 | 3p22 |
|  | TGFBR3 | Transforming growth factor, beta receptor III | 7049 | 1p33-p32 |
|  | TWIST1 | Twist homolog 1 (Drosophila) | 7291 | 7p21.2 |
|  | TWIST2 | Twist homolog 2 (Drosophila) | 117581 | 2q37.3 |
|  | ZEB1 | Zinc finger E-box binding homeobox 1 | 6935 | 10p11.2 |
|  | ZEB2 | Zinc finger E-box binding homeobox 2 | 9839 | 2q22.3 |
|  |  |  |  |  |
| Estrogen signalling | AR | Androgen receptor | 367 | Xq12 |
|  | BRCA1 | Breast cancer 1, early onset | 672 | 17q21 |
|  | CYP19A1 | Cytochrome P450, family 19, subfamily A, polypeptide 1 | 1588 | 15q21.1 |
|  | ESR1 | Estrogen receptor 1 | 2099 | 6q25.1 |
|  | ESR2 | Estrogen receptor 2 (ER beta) | 2100 | 14q23.2 |
|  | MTA1 | Metastasis associated 1 | 9112 | 14q32.3 |
|  | MTA2 | Metastasis associated 1 family, member 2 | 9219 | 11q12-q13.1 |
|  | MTA3 | Metastasis associated 1 family, member 3 | 57504 | 2p21 |
|  | NCOA1 | Nuclear receptor coactivator 1 | 8648 | 2p23 |
|  | NCOA2 | Nuclear receptor coactivator 2 | 10499 | 8q13.3 |
|  | NCOA3 | Nuclear receptor coactivator 3 | 8202 | 20q12 |
|  | PGR | Progesterone receptor | 5241 | 11q22-q23 |
|  |  |  |  |  |
| Angiogenesis | HIF1A | Hypoxia inducible factor 1, alpha subunit (basic helix-loop-helix transcription factor) | 3091 | 14q21-q24 |
|  | VEGFA | Vascular endothelial growth factor A | 7422 | 6p12 |
|  | VEGFB | Vascular endothelial growth factor B | 7423 | 11q13 |
|  | VEGFC | Vascular endothelial growth factor C | 7424 | 4q34.3 |
|  |  |  |  |  |
| Luminal markers | GATA3 | GATA binding protein 3 | 2625 | 10p15 |
|  | KRT7 | Keratin 7 | 3855 | 12q12-q13 |
|  | KRT8 | Keratin 8 | 3856 | 12q13 |
|  | KRT18 | Keratin 18 | 3875 | 12q13 |
|  | KRT19 | Keratin 19 | 3880 | 17q21.2 |
|  |  |  |  |  |
| Basal markers | ACTA1 | Actin, alpha 1, skeletal muscle | 58 | 1q42.13 |
|  | EGFR | Epidermal growth factor receptor (erythroblastic leukemia viral (v-erb-b) | 1956 | 7p12 |
|  | KRT5 | Keratin 5 | 3852 | 12q12-q13 |
|  | KRT6A | Keratin 6A | 3853 | 12q12-q13 |
|  | KRT6B | Keratin 6B | 3854 | 12q12-q13 |
|  | KRT14 | Keratin 14 | 3861 | 17q12-q21 |
|  | KRT17 | Keratin 17 | 3872 | 17q21.2 |
|  | VIM | Vimentin | 7431 | 10p13 |
|  |  |  |  |  |
| Tight junctions | AMOTL1 | Angiomotin like 1 | 154810 | 11q14.3 |
|  | ASAM | Coxsackie- and adenovirus receptor-like membrane protein | 79827 | 11q24.1 |
|  | CGN | Cingulin | 57530 | 1q21 |
|  | CLDN1 | Claudin 1 | 9076 | 3q28-q29 |
|  | CLDN2 | Claudin 2 | 9075 | Xq22.3-q23 |
|  | CLDN3 | Claudin 3 | 1365 | 7q11.23 |
|  | CLDN4 | Claudin 4 | 1364 | 7q11.23 |
|  | CLDN5 | Claudin 5 | 7122 | 22q11.21 |
|  | CLDN6 | Claudin 6 | 9074 | 16p13.3 |
|  | CLDN7 | Claudin 7 | 1366 | 17p13 |
|  | CLDN8 | Claudin 8 | 9073 | 21q22.11 |
|  | CLDN9 | Claudin 9 | 9080 | 16p13.3 |
|  | CLDN10 | Claudin 10 | 9071 | 13q31-q34 |
|  | CLDN11 | Claudin 11 | 5010 | 3q26.2-q26.3 |
|  | CLDN12 | Claudin 12 | 9069 | 7q21 |
|  | CLDN13 | Claudin 13 | NA | NA |
|  | CLDN14 | Claudin 14 | 23562 | 21q22.3 |
|  | CLDN15 | Claudin 15 | 24146 | 7q11.22 |
|  | CLDN16 | Claudin 16 | 10686 | 3q28 |
|  | CLDN17 | Claudin 17 | 26285 | 21q22.11 |
|  | CLDN18 | Claudin 18 | 51208 | 3q22.3 |
|  | CLDN19 | Claudin 19 | 149461 | 1p34.2 |
|  | CLDN20 | Claudin 20 | 49861 | 6q25 |
|  | CLDN21 | Claudin 21 | 53843 | 4q35.1 |
|  | CLDN22 | Claudin 22 | 53842 | 4q35.1 |
|  | CLDN23 | Claudin 23 | 137075 | 8p23.1 |
|  | CLDN24 | Claudin 24 | 100132463 | 4q35.1 |
|  | CXADR | Coxsackie virus and adenovirus receptor | 1525 | 21q21.1 |
|  | EPCAM | Epithelial cell adhesion molecule | 4072 | 2p21 |
|  | ESAM | Endothelial cell adhesion molecule | 90952 | 11q24.2 |
|  | F11R | F11 receptor | 50848 | 1q21.2-q21.3 |
|  | IGSF5 | Immunoglobulin superfamily, member 5 | 150084 | 21q22.2 |
|  | JAM2 | Junctional adhesion molecule 2 | 58494 | 21q21.2 |
|  | JAM3 | Junctional adhesion molecule 3 | 83700 | 11q25 |
|  | MAGI1 | Membrane associated guanylate kinase, WW and PDZ domain containing 1 | 9223 | 3p14.1 |
|  | MAGI2 | Membrane associated guanylate kinase, WW and PDZ domain containing 2 | 9863 | 7q21 |
|  | MAGI3 | Membrane associated guanylate kinase, WW and PDZ domain containing 3 | 260425 | 1p12-p11.2 |
|  | MARVELD2 | MARVEL domain containing 2 | 153562 | 5q13.2 |
|  | MLLT4 | Myeloid/lymphoid or mixed-lineage leukemia (trithorax homolog, Drosophila) | 4301 | 6q27 |
|  | MPDZ | Multiple PDZ domain protein | 8777 | 9p23 |
|  | OCLN | Occludin | 4950 | 5q13.1 |
|  | SYMPK | Symplekin | 8189 | 19q13.3 |
|  | TJP1 | Tight junction protein 1 (zona occludens 1) | 7082 | 15q13 |
|  | TJP2 | Tight junction protein 2 (zona occludens 2) | 9414 | 9q13-q21 |
|  | TJP3 | Tight junction protein 3 (zona occludens 3) | 27134 | 19p13.3 |
|  |  |  |  |  |
| Adherens junctions | ACTN1 | Actinin, alpha 1 | 87 | 14q24.1-q24.2 |
|  | ACTN4 | Actinin, alpha 4 | 81 | 19q13 |
|  | CADM1 | Cell adhesion molecule 1 | 23705 | 11q23.2 |
|  | CADM2 | Cell adhesion molecule 2 | 253559 | 3p12.1 |
|  | CADM3 | Cell adhesion molecule 3 | 57863 | 1q21.2-q22 |
|  | CADM4 | Cell adhesion molecule 4 | 199731 | 19q13.31 |
|  | CDH1 | Cadherin 1, type 1, E-cadherin (epithelial) | 999 | 16q22.1 |
|  | CDH2 | Cadherin 2, type 1, N-cadherin (neuronal) | 1000 | 18q11.2 |
|  | CDH3 | Cadherin 3, type 1, P-cadherin (placental) | 1001 | 16q22.1 |
|  | CDH4 | Cadherin 4, type 1, R-cadherin (retinal) | 1002 | 20q13.3 |
|  | CTNNA1 | Catenin (cadherin-associated protein), alpha 1 | 1495 | 5q31 |
|  | CTNNB1 | Catenin (cadherin-associated protein), beta 1 | 1499 | 3p21 |
|  | CTNND1 | Catenin (cadherin-associated protein), delta 1 | 1500 | 11q11 |
|  | JUP | Junction plakoglobin | 3728 | 17q21 |
|  | NF2 | Neurofibromin 2 (merlin) | 4771 | 22q12.2 |
|  | PFN1 | Profilin 1 | 5216 | 17p13.3 |
|  | PFN2 | Profilin 2 | 5217 | 3q25.1-q25.2 |
|  | PVR | Poliovirus receptor | 5817 | 19q13.2 |
|  | PVRL1 | Poliovirus receptor-related 1 (herpesvirus entry mediator C) | 5818 | 11q23.3 |
|  | PVRL2 | Poliovirus receptor-related 2 (herpesvirus entry mediator B) | 5819 | 19q13.2 |
|  | PVRL3 | Poliovirus receptor-related 3 | 25945 | 3q13 |
|  | PVRL4 | Poliovirus receptor-related 4 | 81607 | 1q22-q23.2 |
|  | VCL | Vinculin | 7414 | 10q22.2 |
|  | ZYX | Zyxin | 7791 | 7q32 |
|  |  |  |  |  |
| PAR Polarity complex | PARD3 | Par-3 partitioning defective 3 homolog (C. elegans) | 56288 | 10p11.21 |
|  | PARD3B | Par-3 partitioning defective 3 homolog B (C. elegans) | 117583 | 2q33.3 |
|  | PARD6A | Par-6 partitioning defective 6 homolog alpha (C. elegans) | 50855 | 16q22.1 |
|  | PARD6B | Par-6 partitioning defective 6 homolog beta (C. elegans) | 84612 | 20q13.13 |
|  | PARD6G | Par-6 partitioning defective 6 homolog gamma (C. elegans) | 84552 | 18q23 |
|  | PRKCI | Protein kinase C, iota | 5584 | 3q26.3 |
|  | PRKCZ | Protein kinase C, zeta | 5590 | 1p36.33-p36.2 |
|  |  |  |  |  |
| Crumbs Polarity complex | CRB1 | Crumbs homolog 1 (Drosophila) | 23418 | 1q31-q32.1 |
|  | CRB2 | Crumbs homolog 2 (Drosophila) | 286204 | 9q33.3 |
|  | CRB3 | Crumbs homolog 3 (Drosophila) | 92359 | 19p13.3 |
|  | INADL | InaD-like (Drosophila) | 10207 | 1p31.1 |
|  | MPP5 | Membrane protein, palmitoylated 5 (MAGUK p55 subfamily member 5) | 64398 | 14q23.3 |
|  |  |  |  |  |
| Scrib Polarity complex | DLG1 | Discs, large homolog 1 (Drosophila) | 1739 | 3q29 |
|  | DLG2 | Discs, large homolog 2 (Drosophila) | 1740 | 11q14.1 |
|  | DLG3 | Discs, large homolog 3 (Drosophila) | 1741 | Xq13.1 |
|  | DLG4 | Discs, large homolog 4 (Drosophila) | 1742 | 17p13.1 |
|  | DLG5 | Discs, large homolog 5 (Drosophila) | 9231 | 10q23 |
|  | LLGL1 | Lethal giant larvae homolog 1 (Drosophila) | 3996 | 17p11.2 |
|  | LLGL2 | Lethal giant larvae homolog 2 (Drosophila) | 3993 | 17q24-q25 |
|  | SCRIB | Scribbled homolog (Drosophila) | 23513 | 8q24.3 |
|  |  |  |  |  |
| LKB1 Polarity regulator | CAB39 | Calcium binding protein 39 | 51719 | 2q37.1 |
|  | CAB39L | Calcium binding protein 39-like | 81617 | 13q14.2 |
|  | STK11 | Serine/threonine kinase 11 | 6794 | 19p13.3 |
|  | STRADA | STE20-related kinase adaptor alpha | 92335 | 17q23.3 |
|  | STRADB | STE20-related kinase adaptor beta | 55437 | 2q33.1 |
|  |  |  |  |  |
| Small GTPase family | CDC42 | Cell division cycle 42 (GTP binding protein, 25kDa) | 998 | 1p36.1 |
|  | RAC1 | Ras-related C3 botulinum toxin substrate 1 (rho family, small GTP binding protein Rac1) | 5879 | 7p22 |
|  | RHOA | Ras homolog gene family, member A | 387 | 3p21.3 |
|  | TIAM1 | T-cell lymphoma invasion and metastasis | 7074 | 21q22.11 |
|  |  |  |  |  |
| Cell-fate decision | ABCG2 | ATP-binding cassette, sub-family G (WHITE),member 2 | 9429 | 4q22 |
|  | ALDH1A1 | Aldehyde dehydrogenase 1 family, member A1 | 216 | 9q21.13 |
|  | ALDH1A3 | Aldehyde dehydrogenase 1 family, member A3 | 220 | 15q26.3 |
|  | BMI1 | BMI1 polycomb ring finger oncogene | 648 | 10p11.23 |
|  | CD24 | CD24 molecule | 100133941 | 6q21 |
|  | CD44 | CD44 molecule (Indian blood group) | 960 | 11p13 |
|  | CDKN1A | Cyclin-dependent kinase inhibitor 1A (p21, Cip1) | 1026 | 6p21.2 |
|  | CDKN1B | Cyclin-dependent kinase inhibitor 1B (p27, Kip1) | 1027 | 12p13.1-p12 |
|  | CDKN2A | Cyclin-dependent kinase inhibitor 2A (melanoma, p16, CDK4) | 1029 | 9p21 |
|  | CDKN2B | Cyclin-dependent kinase inhibitor 2B (p15, inhibits CDK4) | 1030 | 9p21 |
|  | CDKN2D | Cyclin-dependent kinase inhibitor 2D (**p19**, inhibits CDK4) | 1032 | 19p13 |
|  | DLL1 | **Delta**-like 1 (Drosophila) | 28514 | 6q27 |
|  | DLL3 | **Delta**-like 3 (Drosophila) | 10683 | 19q13 |
|  | DLL4 | **Delta**-like 4 (Drosophila) | 54567 | 15q14 |
|  | FZD1 | Frizzled homolog 1 (Drosophila) | 8321 | 7q21 |
|  | FZD2 | Frizzled homolog 2 (Drosophila) | 2535 | 17q21.2 |
|  | FZD3 | Frizzled homolog 3 (Drosophila) | 7976 | 8p21 |
|  | FZD4 | Frizzled homolog 4 (Drosophila) | 8322 | 11q14.2 |
|  | FZD5 | Frizzled homolog 5 (Drosophila) | 7855 | 2q33.3 |
|  | FZD6 | Frizzled homolog 6 (Drosophila) | 8323 | 8q22.3-q23.1 |
|  | FOXA1 | Forkhead box A1 | 3169 | 14q12-q13 |
|  | FOXC1 | Forkhead box C1 | 2296 | 6p25 |
|  | GSK3B | Glycogen synthase kinase 3 beta | 2932 | 3q13.3 |
|  | JAG1 | Jagged 1 | 182 | 20p12.1-p11.23 |
|  | JAG2 | Jagged 2 | 3714 | 14q32 |
|  | MDM2 | Mdm2 p53 binding protein homolog (mouse) | 4193 | 12q14.3-q15 |
|  | NANOG | Nanog homeobox | 79923 | 12p13.31 |
|  | NOTCH1 | Notch 1 | 4851 | 9q34.3 |
|  | NOTCH2 | Notch homolog 2 (Drosophila) | 4853 | 1p13-p11 |
|  | NOTCH3 | Notch homolog 3 (Drosophila) | 4854 | 19p13.2-p13.1 |
|  | NOTCH4 | Notch homolog 4 (Drosophila) | 4855 | 6p21.3 |
|  | NUMB | Numb homolg (Drosophila) | 8650 | 14q24.3 |
|  | POU5F1 | POU class5 homeobox 1 | 5460 | 6p21.31 |
|  | PROM1 | Prominin 1 | 8842 | 4p15.32 |
|  | SOX2 | SRY (sex determining region Y)-box 2 | 6657 | 3q26.3-q27 |
|  | SOX4 | SRY (sex determining region Y)-box 4 | 6659 | 6p22.3 |
|  | SOX9 | SRY (sex determining region Y)-box 9 | 6662 | 17q23 |
|  | TP53 | Tumor protein p53 | 7157 | 17p13.1 |
