# Supplementary material for: Cell Polarity, Epithelial-Mesenchymal Transition, and Cell-Fate Decision Gene Expression in Ductal Carcinoma In Situ
Source: Int J Surg Oncol. 2012 Apr 2;2012:984346. doi: 10.1155/2012/984346 (PMC3335180; doi:10.1155/2012/984346)
Supplement: Supplementary file 2 [file 984346.f2.doc]

Supplementary Table 2**:** Affymetrix probe-sets corresponding to genes entered in the study

| *Official*  *Gene Symbol* | *Entrez*  *Gene ID* | *Affymetrix ID*  (GeneChip U-133A) | *Sensitivity Score* | | *Specificity Score* |
| --- | --- | --- | --- | --- | --- |
| ABCG2 | 9429 | 209735_at | 1 | 1 | |
| ACTA1 | 58 | 203872_at | 1 | 1 | |
| ACTN1 | 87 | 208636_at  208637_x_at  211160_x_at | 1  1  0.73 | 1  1  1 | |
| ACTN4 | 81 | 200601_at | 0.91 | 1 | |
| AKT1 | 207 | 207163_s_at | 1 | 1 | |
| AKT2 | 208 | 203809_s_at  211453_s_at | 1  1 | 1  1 | |
| AKT3 | 10000 | 219393_s_at  212607_at  212609_s_at | 0.73  0.73  0.27 | 1  1  1 | |
| ALDH1A1 | 216 | 212224_at | 0.82 | 1 | |
| ALDH1A3 | 220 | 203180_at | 1 | 1 | |
| AR | 367 | 211110_s_at  211621_at | 1  0.73 | 1  1 | |
| BMI1 | 648 | 202265_at | 0.91 | 1 | |
| BRCA1 | 672 | 211851_x_at  204531_s_at | 1  1 | 1  1 | |
| CAB39 | 51719 | 217873_at | 1 | 1 | |
| CAB39L | 81617 | 221003_s_at | 0.82 | 1 | |
| CADM1 | 23705 | 209030_s_at  209031_at  209032_s_at | 1  0.82  1 | 1  1  1 | |
| CADM3 | 57863 | 211677_x_at  213948_x_at  221921_s_at | 1  1  1 | 1  1  1 | |
| CADM4 | 199731 | 215258_at  215259_s_at | 0.82  0.46 | 1  1 | |
| CD24 | 100133941 | 208650_s_at  208651_x_at  209771_x_at  209772_s_at  216379_x_at  266_s_at | 0.82  0.91  1  0.64  1  1 | 0.89  0.95  1  0.82  1  0.97 | |
| CD44 | 960 | 204489_s_at  204490_s_at  209835_x_at  210916_s_at  212014_x_at  212063_at  217523_at  216056_at | 1  1  1  1  1  1  0.36  0.36 | 1  1  1  1  1  1  1  1 | |
| CDC42 | 998 | 208728_s_at  210232_at  214230_at  208727_s_at | 1  0.73  0.73  1 | 1  1  1  0.95 | |
| CDH1 | 999 | 201130_s_at  201131_s_at | 1  0.64 | 1  1 | |
| CDH2 | 1000 | 203440_at  203441_s_at | 0.82  1 | 1  1 | |
| CDH3 | 1001 | 203256_at | 1 | 1 | |
| CDH4 | 1002 | 206866_at  220227_at | 0.73  0.36 | 1  1 | |
| CDKN1A | 1026 | 202284_s_at | 1 | 1 | |
| CDKN1B | 1027 | 209112_at | 0.73 | 1 | |
| CDKN2A | 1029 | 207039_at  211156_at  209644_x_at | 0.73  0.82  0.82 | 1  1  0.94 | |
| CDKN2B | 1030 | 207530_s_at | 1 | 1 | |
| CDKN2D | 1032 | 210240_s_at | 1 | 1 | |
| CLDN1 | 9076 | 218182_s_at | 0.91 | 1 | |
| CLDN3 | 1365 | 203953_s_at  203954_x_at | 0.46  0.82 | 1  1 | |
| CLDN4 | 1364 | 201428_at | 0.73 | 1 | |
| CLDN5 | 7122 | 204482_at | 1 | 1 | |
| CLDN6 | 9074 | 208474_at | 0.82 | 0.89 | |
| CLDN7 | 1366 | 202790_at | 1 | 1 | |
| CLDN8 | 9073 | 214598_at | 0.82 | 1 | |
| CLDN9 | 9080 | 214635_at | 0.73 | 1 | |
| CLDN10 | 9071 | 205328_at | 1 | 1 | |
| CLDN11 | 5010 | 206908_s_at | 0.91 | 1 | |
| CLDN14 | 23562 | 210689_at | 1 | 1 | |
| CLDN15 | 24146 | 219640_at | 0.91 | 1 | |
| CLDN16 | 10686 | 220332_at | 0.82 | 1 | |
| CLDN17 | 26285 | 221328_at | 1 | 1 | |
| CLDN18 | 51208 | 214135_at  221132_at  221133_s_at | 1  1  1 | 1  1  1 | |
| CRB1 | 23418 | 220522_at | 0.92 | 1 | |
| CTNNA1 | 1495 | 200764_s_at  200765_x_at  210844_x_at | 1  1  0.91 | 1  1  1 | |
| CTNNB1 | 1499 | 201533_at | 1 | 1 | |
| CTNND1 | 1500 | 208407_s_at  208862_s_at  211240_x_at | 1  1  1 | 1  1  1 | |
| CXADR | 1525 | 203917_at | 1 | 1 | |
| CYP19A1 | 1588 | 203475_at | 1 | 1 | |
| DLG1 | 1739 | 202514_at  202515_at  202516_s_at  217208_s_at  215988_s_at | 1  1  1  1  0.55 | 1  1  1  1  1 | |
| DLG2 | 1740 | 206253_at | 0.91 | 1 | |
| DLG3 | 1741 | 207732_s_at  212727_at  212728_at  212729_at | 1  0.91  1  1 | 1  1  1  1 | |
| DLG4 | 1742 | 204592_at  210684_s_at | 0.82  1 | 1  1 | |
| DLG5 | 9231 | 201681_s_at  210469_at | 1  0.55 | 1  1 | |
| DLL3 | 10683 | 219537_x_at | 0.82 | 1 | |
| EGF | 1950 | 206254_at | 0.91 | 1 | |
| EGFR | 1956 | 201983_s_at  201984_s_at  210984_x_at  211550_at  211551_at  211607_x_at | 1  1  1  0.36  0.64  1 | 1  1  1  1  1  1 | |
| EPCAM | 4072 | 201839_s_at | 1 | 0.95 | |
| ERBB2 | 2064 | 210930_s_at  216836_s_at | 1  1 | 1  1 | |
| ESR1 | 2099 | 205225_at  211233_x_at  211234_x_at  211235_s_at  215552_s_at  217190_x_at  211627_x_at  215551_at  217163_at | 1  1  1  1  1  0.55  0.27  0.36  0.27 | 1  1  1  1  1  1  1  1  1 | |
| ESR2 | 2100 | 211120_x_at  211117_x_at  211118_x_at  210780_at  211119_at | 1  1  1  0.64  0.46 | 1  1  1  1  1 | |
| F11R | 50848 | 221664_s_at | 1 | 1 | |
| FOXA1 | 3169 | 204667_at | 0.55 | 1 | |
| FOXC1 | 2296 | 213260_at | 0.64 | 1 | |
| FZD1 | 8321 | 204451_at  204452_s_at | 0.64  0.64 | 1  1 | |
| FZD2 | 2535 | 210220_at | 0.91 | 1 | |
| FZD3 | 7976 | 219683_at | 0.91 | 1 | |
| FZD4 | 8322 | 218665_at | 0.64 | 1 | |
| FZD5 | 7855 | 221254_s_at  206136_at | 1  0.73 | 1  1 | |
| FZD6 | 8323 | 203987_at | 1 | 1 | |
| GATA3 | 2625 | 209602_s_at  209603_at  209604_s_at | 1  0.64  1 | 1  1  1 | |
| GSK3B | 2932 | 209945_s_at | 1 | 0.95 | |
| HIF1A | 3091 | 200989_at | 1 | 1 | |
| HMGA2 | 8091 | 208025_s_at | 1 | 1 | |
| ID1 | 3397 | 208937_s_at | 0.91 | 1 | |
| ID2 | 3398 | 201565_s_at  201566_x_at | 1  0.82 | 1  0.94 | |
| ID3 | 3399 | 207826_s_at | 1 | 1 | |
| ID4 | 3400 | 209291_at  209293_x_at | 0.64  0.82 | 1  1 | |
| INADL | 10207 | 214493_s_at  214705_at | 0.91  1 | 1  1 | |
| JAG1 | 182 | 209097_s_at  209098_s_at  209099_x_at  216268_s_at | 0.82  1  1  1 | 1  1  1  1 | |
| JAG2 | 3714 | 32137_at  209784_s_at | 0.75  0.82 | 1  1 | |
| JAM2 | 58494 | 219213_at | 1 | 1 | |
| JAM3 | 83700 | 212813_at | 1 | 1 | |
| JUP | 3728 | 201015_s_at | 0.91 | 1 | |
| KRT5 | 3852 | 201820_at | 0.82 | 1 | |
| KRT6A | 3853 | 209125_at  214580_x_at  209126_x_at | 0.82  0.64  0.64 | 0.94  0.93  0.29 | |
| KRT6B | 3854 | 213680_at | 0.36 | 1 | |
| KRT7 | 3855 | 209016_s_at | 1 | 1 | |
| KRT8 | 3856 | 209008_x_at | 1 | 0.55 | |
| KRT14 | 3861 | 209351_at | 1 | 0.86 | |
| KRT17 | 3872 | 212236_x_at  205157_s_at | 1  1 | 0.86  0.89 | |
| KRT18 | 3875 | 201596_x_at | 1 | 0.67 | |
| KRT19 | 3880 | 201650_at | 1 | 1 | |
| LLGL1 | 3996 | 206123_at  206124_s_at | 0.91  1 | 1  1 | |
| LLGL2 | 3993 | 203713_s_at | 1 | 1 | |
| MAGI1 | 9223 | 206144_at | 0.91 | 1 | |
| MAGI2 | 9863 | 207702_s_at  209737_at | 0.91  0.82 | 1  1 | |
| MDM2 | 4193 | 217373_x_at  205386_s_at  211832_s_at  217542_at | 1  1  1  0.64 | 1  1  1  1 | |
| MLLT4 | 4301 | 208512_s_at  214939_x_at  215904_at | 1  1  0.46 | 1  1  1 | |
| MPDZ | 8777 | 205079_s_at  213306_at | 1  0.82 | 1  1 | |
| MPP5 | 64398 | 219321_at | 0.73 | 1 | |
| MTA1 | 9112 | 202247_s_at  211783_s_at | 1  1 | 1  1 | |
| MTA2 | 9219 | 203444_s_at | 0.91 | 1 | |
| NANOG | 79923 | 220184_at | 0.91 | 0.95 | |
| NCOA1 | 8648 | 209105_at  209106_at  209107_x_at  210249_s_at | 1  1  0.91  1 | 1  1  1  1 | |
| NCOA2 | 10499 | 205732_s_at  205731_s_at  215605_at | 0.73  0.27  0.73 | 1  1  1 | |
| NCOA3 | 8202 | 207700_s_at  211352_s_at  209062_x_at  209060_x_at  209061_at | 1  1  1  1  0.82 | 1  1  1  1  1 | |
| NF2 | 4771 | 204991_s_at  217150_s_at  211091_s_at  210767_at  218915_at  211092_s_at  211017_s_at | 1  1  1  1  1  1  1 | 1  1  1  1  1  1  1 | |
| NOTCH1 | 4851 | 218902_at | 0.91 | 1 | |
| NOTCH2 | 4853 | 202443_x_at  202445_s_at  210756_s_at  212377_s_at | 0.91  0.73  1  0.91 | 1  1  1  1 | |
| NOTCH3 | 4854 | 203237_s_at  203238_s_at | 0.91  0.55 | 1  1 | |
| NOTCH4 | 4855 | 205247_at | 1 | 0.17 | |
| NUMB | 8650 | 207545_s_at  209073_s_at | 1  1 | 1  1 | |
| OCLN | 4950 | 209925_at | 0.91 | 0.95 | |
| PARD3 | 56288 | 210094_s_at  221280_s_at  221526_x_at  221527_s_at | 1  1  1  1 | 1  1  1  1 | |
| PARD6A | 50855 | 205245_at | 1 | 1 | |
| PARD6B | 84612 | 211907_s_at  214827_at | 0.64  0.27 | 1  1 | |
| PFN1 | 5216 | 200634_at | 1 | 0.86 | |
| PFN2 | 5217 | 204992_s_at | 1 | 1 | |
| PGR | 5241 | 208305_at | 1 | 1 | |
| PI3KCA | 5290 | 204369_at | 0.91 | 1 | |
| POU5F1 | 5460 | 208286_x_at  210265_x_at  210905_x_at  214532_x_at | 0.82  0.73  0.64  0.82 | 0.47  0.39  0.32  0.46 | |
| PRKCI | 5584 | 209677_at  213518_at  209678_s_at | 0.36  0.73  1 | 1  1  1 | |
| PRKCZ | 5590 | 202178_at | 1 | 1 | |
| PROM1 | 8842 | 204304_s_at | 1 | 1 | |
| PTEN | 5728 | 211711_s_at  204053_x_at  217492_s_at  204054_at | 1  1  1  0.73 | 0.68  0.64  0.59  0.88 | |
| PVR | 5817 | 32699_s_at  212662_at  214443_at  214444_s_at  216283_s_at | 1  1  1  1  1 | 1  1  1  1  1 | |
| PVRL1 | 5818 | 208455_at  211845_at  211846_s_at | 0.91  0.64  0.82 | 1  1  1 | |
| PVRL2 | 5819 | 203149_at | 0.91 | 1 | |
| PVRL3 | 25945 | 213325_at | 0.73 | 1 | |
| RAC1 | 5879 | 208640_at  208641_s_at | 1  1 | 1  0.86 | |
| RHOA | 387 | 200059_s_at | 0.82 | 1 | |
| ROCK1 | 6093 | 213044_at  214578_s_at | 0.64  0.55 | 0.86  0.71 | |
| ROCK2 | 9475 | 211504_x_at | 0.82 | 1 | |
| SCRIB | 23513 | 212556_at | 1 | 1 | |
| SIX1 | 6495 | 205817_at | 0.91 | 1 | |
| SMAD2 | 4087 | 203075_at  203076_s_at  203077_s_at | 1  1  1 | 1  1  1 | |
| SMAD3 | 4088 | 205396_at  205397_x_at  205398_s_at  218284_at | 0.91  1  1  1 | 1  1  1  1 | |
| SMAD4 | 4089 | 202526_at  202527_s_at | 0.73  1 | 1  1 | |
| SMAD6 | 4091 | 209886_s_at  207069_s_at | 1  0.82 | 1  1 | |
| SMAD7 | 4092 | 204790_at | 0.73 | 1 | |
| SMURF1 | 57154 | 212666_at  212668_at  215458_s_at | 1  0.91  1 | 1  1  1 | |
| SNAI1 | 6615 | 219480_at | 0.73 | 1 | |
| SNAI2 | 6591 | 213139_at | 0.73 | 1 | |
| SOX2 | 6657 | 213721_at  213722_at | 0.82  0.27 | 0.91  1 | |
| SOX4 | 6659 | 201417_at  213668_s_at  201418_s_at  201416_at | 0.91  0.73  0.73  0.46 | 1  1  0.94  1 | |
| SOX9 | 6662 | 202936_s_at  202935_s_at | 0.73  0.55 | 1  1 | |
| STK11 | 6794 | 41657_at  204292_x_at | 1  1 | 1  1 | |
| STRADA | 92335 | 52169_at  221554_at | 1  1 | 1  1 | |
| SYMPK | 8189 | 32402_s_at  202339_at | 1  1 | 1  1 | |
| TCF3 | 6929 | 209152_s_at  209153_s_at  213730_x_at  213811_x_at  215260_s_at  209151_x_at  210776_x_at | 1  1  0.91  1  1  1  1 | 1  1  1  1  1  1  0.95 | |
| TGFB1 | 7040 | 203084_at  203085_s_at | 0.55  1 | 1  1 | |
| TGFB2 | 7042 | 220407_s_at  209908_s_at  209909_s_at | 1  0.91  0.91 | 1  1  1 | |
| TGFB3 | 7043 | 209747_at | 0.55 | 1 | |
| TGFBR1 | 7046 | 206943_at | 0.64 | 1 | |
| TGFBR2 | 7048 | 207334_s_at  208944_at | 1  0.64 | 1  1 | |
| TGFBR3 | 7049 | 204731_at | 1 | 1 | |
| TIAM1 | 7074 | 213135_at  206409_at | 1  1 | 1  1 | |
| TJP1 | 7082 | 202011_at  214168_s_at | 1  1 | 1  1 | |
| TJP2 | 9414 | 202085_at | 1 | 1 | |
| TJP3 | 27134 | 213412_at  35148_at | 1  1 | 0.95  1 | |
| TP53 | 7157 | 201746_at  211300_s_at | 1  1 | 1  1 | |
| TWIST1 | 7291 | 213943_at | 0.82 | 1 | |
| VCL | 7414 | 200930_s_at  200931_s_at | 0.73  0.91 | 1  1 | |
| VEGFA | 7422 | 210512_s_at  210513_s_at  211527_x_at  212171_x_at | 1  1  0.91  1 | 1  1  1  1 | |
| VEGFB | 7423 | 203683_s_at | 0.91 | 1 | |
| VEGFC | 7424 | 209946_at | 1 | 1 | |
| VIM | 7431 | 201426_s_at | 1 | 1 | |
| ZEB1 | 6935 | 212764_at  210875_s_at  212758_s_at | 1  1  1 | 1  1  1 | |
| ZEB2 | 9839 | 203603_s_at | 1 | 1 | |
| ZYX | 7791 | 200808_s_at  215706_x_at | 1  1 | 1  1 | |
